# Nanomedicine strategies for central nervous system (CNS) diseases

**DOI:** 10.3389/fbiom.2023.1215384

**Published:** 2023-08-10

**Authors:** Shreya Nagri, Olivia Rice, Yupeng Chen

**Affiliations:** Department of Biomedical Engineering, University of Connecticut, Storrs, CT, United States

**Keywords:** nanomedicine, blood-brain barrier, central nervous system disease, Alzheimer’s, Parkinson’s, multiple sclerosis, cancer, glioblastoma

## Abstract

The blood-brain barrier (BBB) is a crucial part of brain anatomy as it is a specialized, protective barrier that ensures proper nutrient transport to the brain, ultimately leading to regulating proper brain function. However, it presents a major challenge in delivering pharmaceuticals to treat central nervous system (CNS) diseases due to this selectivity. A variety of different vehicles have been designed to deliver drugs across this barrier to treat neurodegenerative diseases, greatly impacting the patient’s quality of life. The two main types of vehicles used to cross the BBB are polymers and liposomes, which both encapsulate pharmaceuticals to allow them to transcytose the cells of the BBB. For Alzheimer’s disease, Parkinson’s disease, multiple sclerosis, and glioblastoma brain cancer, there are a variety of different nanoparticle treatments in development that increase the bioavailability and targeting ability of existing drugs or new drug targets to decrease symptoms of these diseases. Through these systems, nanomedicine offers a new way to target specific tissues, especially for the CNS, and treat diseases without the systemic toxicity that often comes with medications used currently.

## 1 Introduction

**FIGURE 1:**
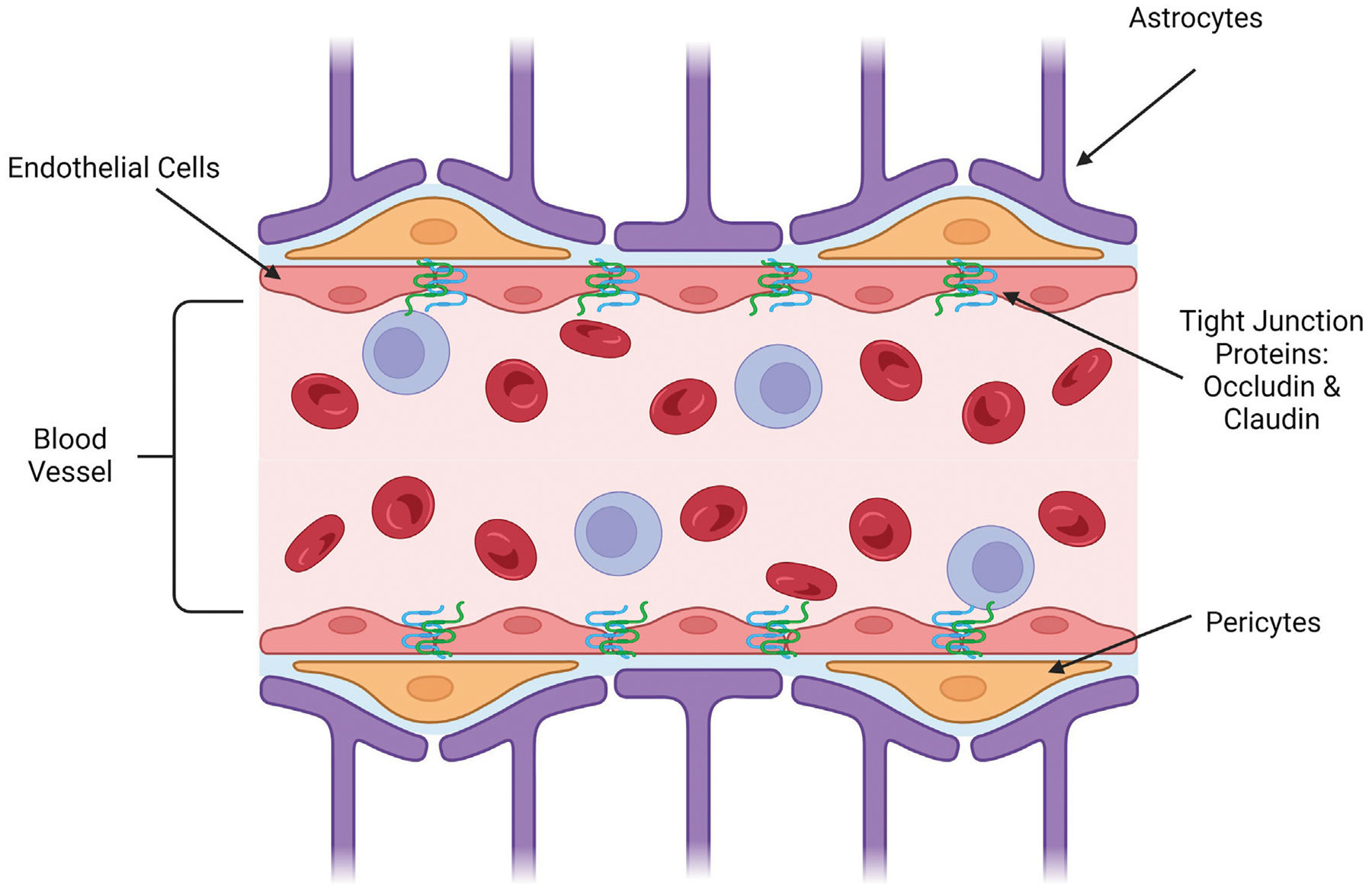
Longitudinal representation of blood-brain barrier anatomy. (Image created using Biorender.com. Inspired by (Sofroniew and Vinters, 2010; Jo et al., 2013; Cabezas et al., 2014; Daneman and Prat, 2015; Serlin et al., 2015; Herland et al., 2016; Stephen and Barrand, 2016; Dong, 2018; Gastfriend et al., 2018; Ayloo and Gu, 2019; Kim et al., 2019; Kadry et al., 2020; Galea, 2021; Rice et al., 2022)).

The blood-brain barrier (BBB) is a crucial physiological characteristic of the human nervous system that creates the division between the blood circulating through the body and the cerebrospinal fluid (CSF) of the brain and spinal cord (Daneman and Prat, 2015). It plays a crucial role in the immune response and regulation of CSF by preventing the passage of many molecules and pathogens that can circulate in the blood (Kadry et al., 2020). A barrier like this allows for a difference in the concentration of beneficial molecules, like glucose, potassium ions, and sodium ions, between the blood and cerebrospinal fluid (Stephen and Barrand, 2016). It also allows for additional immune protection of the brain by creating another barrier that pathogens must pass through to affect the entire body, protecting one of the most important organs in the body (Galea, 2021). This barrier is highly selective mainly due to the presence of tight junctions, which work as intercellular zippers, formed between the endothelial cells that surround the blood vessel connecting to the BBB (Serlin et al., 2015; Ayloo and Gu, 2019). This prevents the paracellular transport of molecules around cells, forcing molecules to transcytose through the endothelial cells to gain access to the CSF (Rice et al., 2022). Although these endothelial cells are the primary cell type present in the BBB, the nearby blood vessel is also surrounded by pericytes and astrocytes, which play a role in the regulation of molecule flow through the BBB (Herland et al., 2016). Pericytes are cells wrapped in a basement membrane that surround the endothelial cells of the blood vessel that encourage barrier genesis by regulating tight junction morphology, transcytosis, and expression of leukocyte adhesion molecules, which mediate the adhesion of cells (Gastfriend et al., 2018). These cells enhance the tightness of the junctions between endothelial cells, thus regulating which molecules will be able to permeate the BBB (Jo et al., 2013). Astrocytes are a type of glial cell that help to hold nerve cells in place and allow them to access nutrients (Kim et al., 2019). Anatomically, astrocytes also have projections, called feet, that can send signals to nearby cells to modulate activity (Sofroniew and Vinters, 2010). Astrocyte projections are also wrapped in the pericytic basement membrane to modulate the permeability of the BBB by manipulating the integrity of tight junctions (Cabezas et al., 2014). These three cell types are the main components of the BBB that function as the selective barrier that must be overcome in drug delivery applications (Dong, 2018). Figure 1 shows the anatomy of the BBB that must be overcome for delivery.

Tight junctions between endothelial cells are formed by a variety of interacting proteins and the cytoskeletons of the cells themselves, creating a barrier that prevents paracellular transport (Anderson and Van Itallie, 2009; Alahmari, 2021). Two of the main proteins involved in forming tight junctions are occludin and claudin (Beeman et al., 2012). These proteins are characterized as transmembrane sealing proteins that are specialized to prevent paracellular transport by creating a stronger tight junction between cells (Heinemann and Schuetz, 2019). Other proteins involved in the tight junctions of the BBB are integral membrane proteins and junctional adhesion molecules, which both serve to further bind the cell membranes together while participating in cell signaling and responding to signals from astrocyte feet (Stamatovic et al., 2016). While the selectivity of the BBB is essential to the immune protection of the brain, regulation of ions across compartments, and fluid regulation, it also represents a large challenge in research for drug delivery to treat diseases of the brain (Achar et al., 2021). Drugs that aim to treat CNS diseases must be able to reach the brain, their target tissue, or cell type, ultimately treating the disease at its source (Khawli and Prabhu, 2013). However, this involves crossing the BBB. Many drugs produced to treat the brain are not able to cross the BBB due to several factors including size, charge, and hydrophobicity that are often complicated by the structures necessary for the drug to function as originally designed (Krol, 2012). One of the avenues being explored to overcome this challenge is the integration of nanomedicine techniques for the treatment of neurodegenerative disease (You and Sabel, 2020; Li et al., 2021).

**FIGURE 2:**
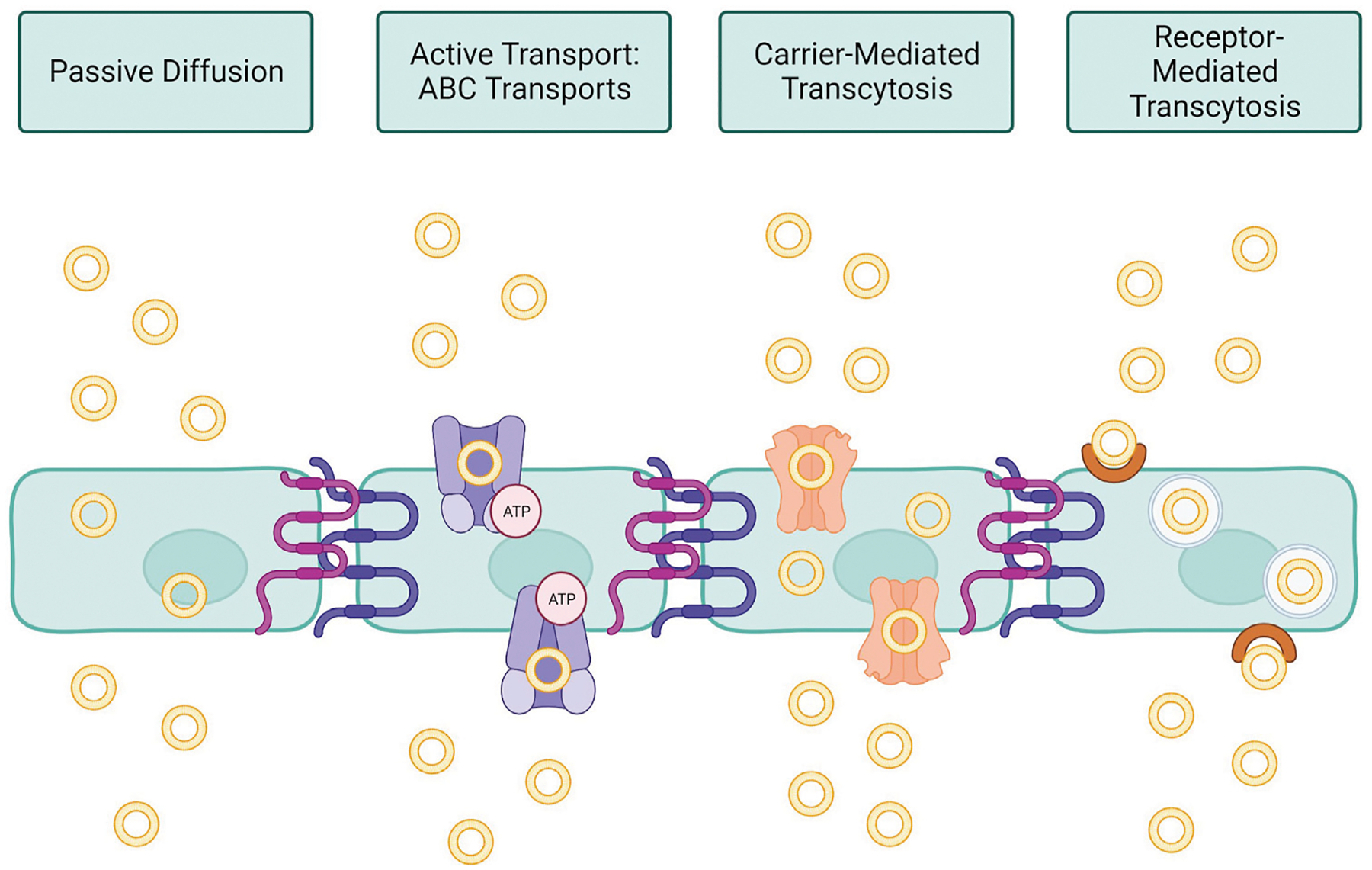
Nanomedicine pathways across BBB endothelial cells. Passive diffusion requires no energy or specific targeting, this pathway is only utilized by small molecules or ions essential to proper brain function. Active transport requires ATP in order to induce the uptake of nanoparticles by ATP-binding cassette (ABC) transporters. Carrier-mediated transcytosis functions as a solute-carrier complex that allows transport across cells. Receptor-mediated transcytosis specifically targets a known receptor on the surface of brain endothelial cells, promoting uptake into the cell within an endosome. (Image created using Biorender.com. Inspired by (Brown et al., 2020; Laura Del AmoCano et al., 2021)).

Nanomedicine is a sector of clinical medicine research that focuses on developing nanoparticles that can be used to deliver therapeutics and diagnostics (Lee Ventola, 2012). Nanoparticles (NPs) are a highly engineerable class of materials on the nanoscale that can be used to deliver therapeutics into cells with high targeting ability, high efficiency, and low toxicity (Murthy, 2007). These qualities are important for a delivery system because they allow for greater efficacy in the clinical setting and fewer undesirable systemic side effects for the patient (Sun et al., 2019). They can also allow for hydrophobic drugs to be introduced into the body, which is otherwise an unfavorable process (Song et al., 2011). For drug delivery applications, an NP is optimally around 50 nm in size, with a functional size range between 10 and 500 nm, and due to this, they function on the same scale as many biological processes, adding to their biocompatibility (Hoshyar et al., 2016; Rizvi and Saleh, 2018). Structurally, NPs can be engineered to allow for high drug loading and activity, resulting in enhanced delivery targeting (Sun et al., 2020). Additional customizable options include the surface charge of the particle, the functional groups attached, the backbone of the complex, and its size depending on the drug that it is interacting with and the final target tissue of the drug (Zhang and Saltzman, 2013; Sanità et al., 2020). Oftentimes, these NPs are used to deliver hydrophobic molecules or gene therapy that would otherwise be either bioincompatible or difficult to distribute throughout the body (such as gene therapy or siRNAs) (Zhou et al., 2020a; Chen and Yu, 2012; Zhou et al., 2020b). Different applications require different engineering to be able to fit the situation. There are four main avenues for NPs to cross the BBB that are currently being investigated (Laura Del AmoCano et al., 2021; Brown et al., 2020). The first is passive diffusion, which involves creating a small hydrophobic molecule that is capable of crossing through the BBB and into the brain subsequently. The next avenue is active transport by binding to ABC transporters, which metabolize ATP to pump molecules through endothelial cells and into the brain. The third avenue is carrier-mediated transcytosis, which takes advantage of existing carriers on endothelial cells to complex with delivery systems and transfect the therapeutic. Finally, the fourth avenue is receptor mediated endocytosis, which uses a targeting ligand on the system to be taken up by the endothelial cell and ejected into the brain for delivery. These avenues are shown in Figure 2. Currently, polymer-based and liposome-based NPs have been reported as successful nanoscale systems for drug delivery to many parts of the body, including the heart and liver (Sun et al., 2022; Lomis et al., 2021). In addition to drug delivery applications, inorganic nanoparticles, typically gold-NPs, are being used in medical imaging to help visualize images more clearly (Li and Chen, 2015). These nanoparticles help to render images clearly due to their ability to absorb energy and reflect it back without significant loss in their fundamental properties or structure (Dykman and Khlebtsov, 2011; Yau et al., 2019a). This review will explore the features of nanomedicine that make it an ideal drug delivery method for transporting therapeutics across the BBB and the current therapies that are being developed and tested for the treatment of CNS diseases.

### 1.1 Historical background of nanomedicine

The field of nanomedicine has had many recent groundbreaking advancements. However, throughout history, scientists have been exploring the capabilities of nanomaterials, which has impacted the way the field is viewed today. Prior to experimenting with nanomaterials, scientists had to develop the technology to visualize these materials, which was developed in 1903 (Masters, 2020). Scientists were able to view particles under 2 nm using an ultramicroscope, which then sparked interest throughout the 20th century in the research of nanoscale materials (Yan Lee and Wong, 2011). When transmission electron microscopy (TEM) was invented in 1931, these structures were able to be viewed in much higher resolution, allowing for a greater understanding of the structure of the materials (Frontiers, 2018). With this and new technology being developed, it was then possible to regularly view objects at the nanoscopic scale.

Nanotechnology as an idea was first famously outlined in 1959 by Dr. Richard P Feynman, a Nobel Prize-winning theoretical physicist, who gave a lecture introducing the ideas of nanotechnology as a new way to store information and create new technology at the molecular level (Bayda et al., 2019; Pitkethly, 2008). However, the term “nanotechnology” was not firmly coined until 1986, when one of the first textbooks *Engines of Creation: The Coming Era of Nanotechnology* by K. Eric Drexler on this research area was published (Drexler, 1986). This textbook details structures made from individual atoms that self-manipulate and therefore self-replicate and assemble, which is the basis for nanomedicine technology today (Eric drexler, 1992). In combination with the publishing of these textbooks and the influx of research, along with a greater understanding of biochemistry, the use of nanomaterials in medicine development became a major area of interest (Germain et al., 2020). In the 1970s, monoclonal antibodies were synthesized for the first time, furthering interest in the idea of using a nano-scale vehicle to deliver therapeutics to cells (Kohler and Milstein, 1975). The term “nanomedicine,” despite so much research in the area, however, was not coined until 1991 in Drexler’s next textbook, titled “Unbounding the Future: the Nanotechnology Revolution” (Drexler et al., 1991). Currently, liposomes, biocompatible polymers, and micelles are the subject of most research in the field because of their small size and compatibility with the bloodstream (Domingues et al., 2022). The first nanotherapeutic to be approved by the FDA was a PEGylated adenosine deaminase enzyme, which was approved in 1990 and manufactured by Sigma Tau, sold as Adagen, to treat patients with severe combined immunodeficiency (SCID) (ScienceDirect, 2018). This drug was closely followed by the 1995 FDA approval of Doxil, sold by Baxter, which is a PEGylated nanoliposome used in targeted chemotherapy treatments (Barenholz, 2012). Today, there are far more FDA-approved nanotherapeutics on the market for a variety of different diseases, including cancer, which provide promise for the future of medical therapeutics and directions for further research.

### 1.2 Current FDA-approved nanotherapeutics

There are a variety of current FDA-approved nanotherapeutics that are used to treat various conditions. This being said, the vehicles used by these therapies are limited, as many utilize variations of the same technology to fit the chemistry of their therapeutic to be delivered (Patra et al., 2018). The two main types of nanotherapeutics on the market today are liposomal nanoparticles and PEGylated polymers (Anselmo and Mitragotri, 2019). Both particles have had great success in delivering therapeutics into cells and are highly engineerable to be applicable to several different diseases and drug targets using side chains to alter surface charge and size (Abd Ellah and Abouelmagd, 2017). These distinct types of vehicles are being used by different companies under trade names, but rely on the same mechanism to deliver drugs into tissues and cells (Farjadian et al., 2019). Liposomal nanoparticles are often taken into cells through extravasation, or leakage, from the bloodstream into the interstitial space, which allows for the passive transport of these complexes to their intended targets (Amarandi et al., 2022). Targeting of these NPs is achieved by altering surface chemistry, including ligands and side chains to alter the receptors that the liposomes can bind to, therefore altering behavior (Malam et al., 2009). PEGylated polymers are able to deliver drugs across the cell membrane by interacting with proteins within the body itself, which then help to facilitate transcytosis of the NP complexed with the drug (JungSuk et al., 2016). By utilizing a PEG coating, it ensures that the individual NPs do not interact with each other and have better targeting ability, as they are prone to self-adhering and aggregating (Shi et al., 2021; Bi et al., 2006). If this happens, the NP complex is too large to enter the cell and administer the drug (Rajan et al., 2006). Table 1 summarizes common side chains that are used to functionalize nanoparticles for delivery. Other types of nanotherapeutics that are FDA-approved are some acetate particle vehicles, which can often form polymers themselves to deliver efficiently after subcutaneous injection, and interleukin which is related to naturally occurring proteins in the blood that can be circulated to their intended target through the bloodstream (Sartor, 2003; Green et al., 2007). Although there has been great progress in the development and approval of nanomedicine therapeutics, there is still significant research being conducted, specifically in the neurological space.

## 2 Current nanomedicine research and therapeutics in neurodegenerative disease

Currently, research is being conducted in order to investigate the use of nanotherapeutics for the treatment of various diseases that affect the CNS. Neurodegenerative diseases research is of significant interest, as the impact is widely felt around the world (Jagaran and Singh, 2021). These are also often diseases that present challenges in drug delivery due to changes in brain anatomy that occur during the course of the illness. Specifically, this section of the paper will cover research pertaining to Alzheimer’s Disease, brain cancers, specifically focusing on glioblastomas, Parkinson’s Disease, and multiple sclerosis.

### 2.1 Alzheimer’s disease (AD)

Alzheimer’s disease is a specific form of dementia that progresses from mild memory loss to full cognitive impairment, limiting the patient’s ability to complete daily tasks. It is the most common form of dementia, affecting more than 5.8 million people in the United States alone and worldwide estimates are over 24 million (alz, 2023). These numbers are only increasing over time with projections showing that total patient numbers may triple by 2060 (CDC, 2022). The risk of developing this disease increases over time, however, early onset can occur due to genetic factors. The cause of AD is not fully understood, but studies have shown that the disease is physically characterized by an increase in the number of amyloid-β plaques (Aβ), neurofibrillary tangles (NFTs), and inflammation of the brain. These pathogenic measures can also be caused by normal aging, so diagnosis currently hinges on symptoms, such as memory loss, decreased judgment, and changes in mood (DeTure and Dickson, 2019). Aducanumab (Aduhelm), developed by Biogen and Eisai, is currently the only FDA approved therapy for this disease, which targets the protein Aβ using monoclonal antibodies to prevent the growth of Aβ plaques in patients that are associated with AD. However, this drug is only approved to use for those who have been recently diagnosed with AD and are only suffering from mild symptoms of the disease (National Institute on Aging, 2022a). Other FDA approved drugs, including donepezil, rivastigmine, and memantine, aimed at treating AD patients only manage symptoms of the disease and slow the pathogenic progression, however none of these options offer a cure for the condition (Cui et al., 2018; Devanand et al., 2018). Current nanotherapeutic research involves managing the physiological changes that accompany AD to slow or reverse the effects of the disease.

#### 2.1.1 Current NP-based Alzheimer’s disease research

**FIGURE 3:**
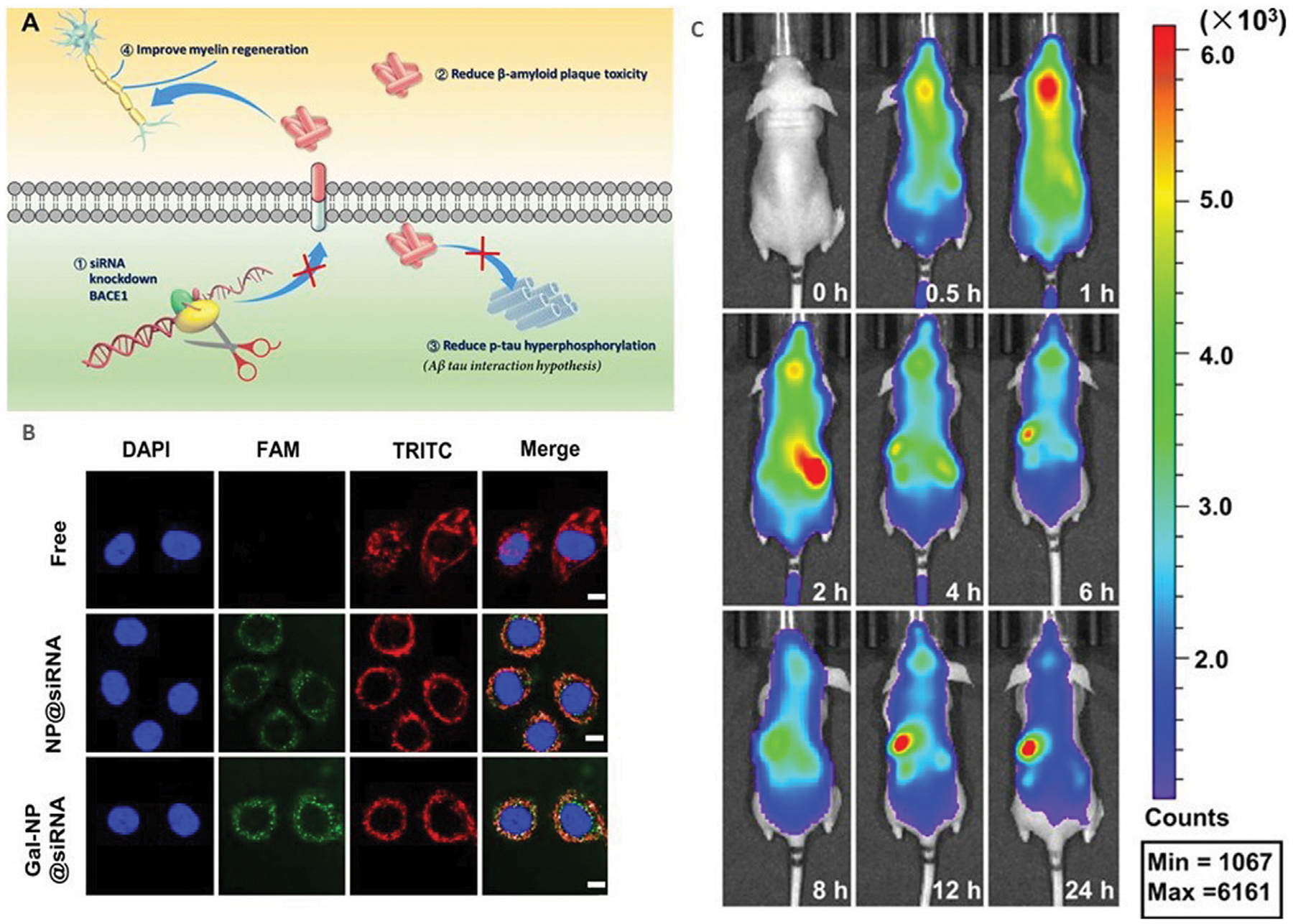
Current research in AD nanotherapeutics. **(A)** siBACE1 therapy mechanistic schematic. **(B)** *In vitro* cellular uptake of the Gal-NP loaded with siRNA in Neuro-2a cells using Cy5 to label the NPs. The siRNA localization is traced in green. **(C)** *In vivo* time lapse biodistribution. Reproduced from (Zhou Yutong et al., 2020).

Currently, scientists are developing functionalized NPs in order to target AD specific pathologies and effectively deliver their drug across the BBB. Figure 3 shows the mechanism of action that many treatments target to decrease physiology associated with AD, along with results of various studies that have shown successful transfection to brain tissue. One study, conducted by Zhou et al., studied the use of an siRNA drug with a polymeric NP, specifically a galactose-modified poly(ethylene glycol)-block-poly[N-(3-methacrylamidopropyl)] guanidium (GalPEG-b-P(Gu)) vehicle, to block the activity of the BACE1 enzyme in the brain that increases the accumulation of Aβ (Zhou Yutong et al., 2020; Cole and Vassar, 2007). This system offers an effective, nonviral, BBB-penetrable way to decrease the activity of this enzyme, which has been impossible previously at a size of 118 nm in a spherical morphology. siRNAs are short pieces of RNA that are able to attack specific targets, including genetic material or proteins, in order to impact the expression of specific genes and therefore expressed proteins. The complex was administered glycosylated, which gave it “triple interaction” stabilization to cross the BBB after going through the blood stream, and fluorinated medicines were also found to be more stable during the administration process. In order to test the delivery and efficacy of this complex, an *in vitro* using a Transwell model and an *in vivo* mouse model were utilized. Results of these tests were measured using stability studies of the NP complex itself, the biodistribution of a tagged version of the drug after administration through tissue studies, cytotoxicity, and the qualitative behavior of the mice. Using these measures, the drug was found to be effective in decreasing symptoms and crossing the BBB, indicating a potential future avenue for drug investigation.

A liposome-based therapy for AD was researched by Arora et al.to develop an alternative method to cross the BBB and target another enzyme involved with Aβ, apolipoprotein E (Arora et al., 2021). Apolipoprotein E (ApoE2) is a protein that plays an important role in the removal of Aβ synthesizing proteins and is made by astrocytes located around the BBB (Li et al., 2020). In this study, the gene encoding this protein was introduced to the brain using a liposome NP. The NP was surface functionalized using a GLUT-1 targeting ligand mannose and a cell penetrating peptide (CPP) to increase brain targeting and cellular uptake. These NPs were spherical and were found to be around 172 nm with a zeta potential of 19 mV. This NP was only tested *in vitro* using a Transwell model, both as a single culture and a coculture. The cells used in these experiments were primary cells from rats, both astrocytes and bEnd-3 cells, which are both integral parts of the rat BBB anatomy. For both models, transendothelial electrical resistance (TEER), which is the most common model validation technique for *in vitro* BBB experiments, was recorded to measure the barrier integrity, with experiments being conducted once the barrier integrity was optimized (Vigh et al., 2021). The coculture experiment had a peak TEER of around 175 Ω/cm^2^ at which point the liposome was successfully delivered across the barrier. Results showed that the liposome system helped to improve transport of the gene across the BBB by preventing endonuclease digestion and improving cytotoxicity of the drug itself. In mouse models, the pApoE2 genes were shown to be expressed after treatment, indicating therapeutic potential of this delivery method in the treatment of AD.

In order to develop a more targeted approach, researchers have been investigating NP formulations that focus on specific properties of the BBB and other pathological features of an AD brain to create a more effective drug. In a study by Cesur et al., microbubbles were exploded to create a complexed NP with portions of the FDA approved therapy donepezil and BiFeO_3_, making the NP electrically sensitive to control release, with a base of polyvinyl alcohol (PVA) (Cesur et al., 2022). It had a size of around 164 nm. This NP was explored to increase the efficacy of donepezil while decreasing side effects, and the frequency of administration typically needed (Knowles, 2006). This complex was biocompatible and found to be effective in creating an electrically active NP that could be delivered across the BBB, creating new avenues to control the specificity of a drug. Another approach, studied in Ribeiro et al., used mesoporous silica to create nanocontainers used to store curcumin, which is a natural drug which has anti-inflammatory and anti-amyloid accumulation activity, that were then dispersed in a thermally active hydrogel (Ribeiro et al., 2022; Tang and Taghibiglou, 2017). The NP was characterized to be spherical with a size of around 158.10 nm and a charge of around −16.73 mV. This NP was tested *ex vivo* in cells, using a microfluidic model as detailed in Rodero et al., and *in vivo* in mouse models to determine the cytotoxicity, which was insignificant, and the efficacy in AD expressing mice *versus* controls (Rodero et al., 2018). Microfluidics allows for testing more closely related to the actual BBB than a Transwell plate because of the dynamic media flowing through the model, especially made possible through tissue chips (Yau et al., 2023). The administration of this treatment was found to reverse the cognitive deficiencies of these mice, which opens another potential drug investigation avenue.

These therapies show that depending on the drug target, NP material, and surface functionalization can be modified to develop an effective delivery and treatment option for AD. As the knowledge of AD progression increases, numerous potential avenues for the use of nanotherapeutics will expand. NPs can be customized to complex with many different drugs and adapted for different targets, which shows that they are applicable to future drug development.

#### 2.1.2 AD therapeutics and diagnostics in clinical trials

Currently in the United States, there is only one AD diagnostic based on nanomedicine that is in clinical trials that is registered with the government, which is a diagnostic known as ADx-001. This trial is being run by the biopharmaceutical company Alzeca Biosciences, Inc., which is based in Houston, Texas and is investigating a proof-of-concept in humans of their contrast agent ADx-001, which is a lipid-PEG conjugated styryl-pyrimidine. This drug is used to visualize Aβ plaques that form in the brain, providing an avenue to early diagnosis for AD and dementia because it has been shown that Aβ plaques predate symptoms and cause the formation of neurofibrillary tangles. Patients are monitored using MRI scans that are sensitive to specific anatomical changes that happen with AD and can show its progression in patients while they are still alive. The NP is made from the imaging molecule Gd(III)-DSPE-DOTA sodium salt complexed with ET3–73 which targets amyloid plaque specifically. Since this trial is in Phase 1 with the FDA, researchers are measuring immediate adverse reactions to the drug that may raise concern for its safety and are looking longitudinally over 2 years to observe any differences in the anatomical structure of patients’ brains (Andrew et al., 2020).

### 2.2 Cancer

Brain tumors, or uncontrolled cell growth originating from the brain, is a prevalent type of cancer that can have a variety of effects depending on where the tumor is located. Brain and nervous system tumors affect 30 out of 100,000 adults in the United States, making this a prevalent issue (Miller et al., 2021). These types of tumors are dangerous because they can put pressure on healthy brain tissue, causing its function to be impaired, or even causing neighboring tissues to become cancerous and spread quickly (hopkinsmedicine, 2022). One of the most heavily researched brain cancers and most common is glioblastoma multiforme, an aggressive type of brain cancer that is formed from astrocytes (Ray-Chaudhury and Ray, 2010). Treatment of these cancers is challenging because the tumor is very malignant and it is most common in older adults, who may not be able to tolerate aggressive chemotherapy (Dain and Zhu, 2023). The current treatment plan for someone with glioblastoma cancer includes chemotherapy, radiation therapy, surgery to excise the tumor, experimental clinical trials, tumor treating fields (TTF) therapy, or palliative care, however survival rates are still very low (mayoclinic, 2022a). These treatments also come with the potential for devastating side effects that occur as a result of these drugs having effects throughout the body and not just in areas affected by cancer. Nanotherapeutics, therefore, are an attractive area of research for future cancer treatments because of their ability to be targeted to a particular tissue and deliver precise, effective treatments. Current research is investing in the use of nanotherapeutics to be able to deliver targeted therapy to cancerous tissues to avoid the major negative systemic effects of many of these current treatments and combat the tumor more effectively.

#### 2.2.1 Current NP-based glioblastoma research

Scientists have been centering glioblastoma research around the use of NPs that are able to change conformation in response to a variety of different pathogenic factors of tumors in order to achieve more targeted drug delivery. Tumor microenvironments often have altered pHs or other conditions that can be exploited to target specific areas of the brain and avoid having the systemic effects that current treatments, such as chemotherapy and radiation, have (Barar and Omidi, 2013). These environmental changes often affect how drugs and other delivery systems function, so designing a vehicle that can adapt to the change in environment is important to effective therapy (Jayasuriya et al., 2016). This is done to deliver a variety of drugs thought to help reduce tumor size, or to deliver chemotherapy drugs to a more targeted location. Another common experimental drug to be delivered are proteins, which are being highly investigated because they have high specificity and specific function in cells (Yau et al., 2021a).

A study by Guo et al. showed that a thiolated paclitaxel-oligo with an angiopep-2 unit, or a modified protein NP, can be used to complex with a variety of different drugs and use its ability of size changing to be able to cross the BBB effectively and target only the tumor tissue (Guo et al., 2020). The NPs only self-assemble into the active drug form when they are in the acidic tumor microenvironment, thus allowing targeted delivery of the drug and avoiding systemic effects based on pH. This method was tested both *in vitro* using fluorescence and *in vivo* in mouse models and found to be effective. Using the angiopep-2 peptide side chain here shows the efficacy of side chain manipulation to functionalize an NP for its intended therapeutic goal.

**FIGURE 4:**
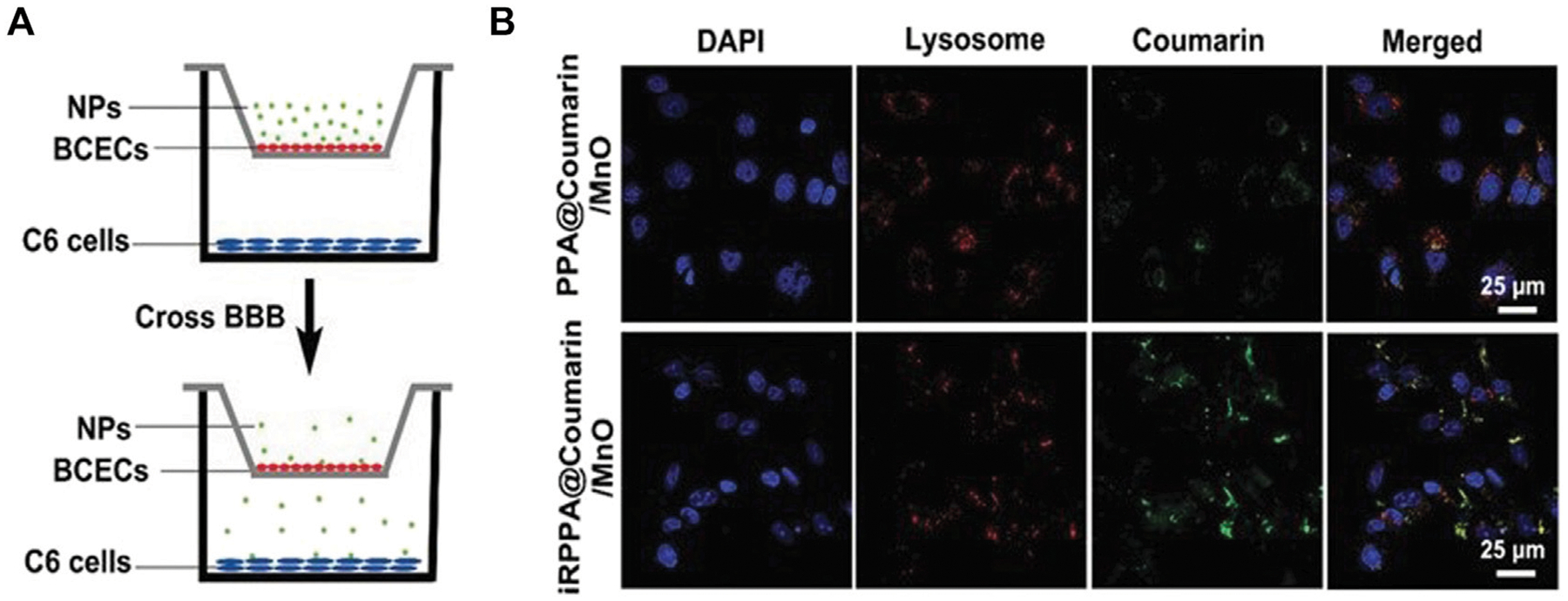
*In vitro* BBB delivery for the treatment of cancer. **(A)**. *In vitro* Transwell model schematic displaying the experimental design for NP delivery. Reproduced from (Tan et al., 2020). **(B)**. Confocal imaging results from the *in vitro* Transwell experiment of the cellular uptake of the NPs into the C6 cancer cells. Reproduced from (Tan et al., 2020).

This strategy of modifying NPs for the delivery of drugs specifically to tumors is also being used in other research. Wang et al. developed a pH sensitive polymeric NP that delivers a antiprogrammed death-ligand 1 (a cancer antibody) across the BBB as tested in a Transwell tissue model (Wang Hairong et al., 2022). The NP is made from p-2-methacryloyloxyethyl phosphorylcholine-co-p-poly(ethylene glycol) methacrylate (pMPC-co-pPEGMA) and exhibits spherical morphology with a diameter around 45 nm. This experiment transfected pMPC-co-pPEGMA, which disassembled at high pHs to release immune checkpoint blockade (ICB) antibodies across the barrier successfully by interacting with endothelial cell surfaces for transfection and triggering an antitumor immune response. The system was then tested in a mouse model, which also showed successful delivery using red tagged mRNA as measured in a confocal microscope, which is the standard for visualization. mRNA can also be detected through other methods, including chemically through rtPCR or through its interactions with other molecules, which has been utilized previously in other nanoparticle studies and has the potential to be used in future CNS studies (Li et al., 2022). Another study by Tan et al. investigated the use of an NP made from oleic manganese monoxide contained in micelles made from polyethylene glycol-poly(2-diisopropylamino)ethyl methacrylate (PEG-PDPA) containing temozolomide (TMZ) and the guiding peptide internalizing arginine-glycine-aspartic acid to cross the BBB and reach the brain (Tan et al., 2020). The NP had a dodecahedron crystal shape and had a size of around 71 nm with a zeta potential of +4.3 mV. This NP was successful at passing through both the *in vitro* Transwell model of the BBB and the *in vivo* mouse model as measured using MRI. The complex was able to successfully enter the glioma and respond to the tumor microenvironment, releasing manganese, O_2_ and TMZ by inducing apoptosis in cancer cells and causing oxidative stress. TMZ is a drug that was derived from various common chemotherapies to combat gliomas and complex with this NP (Fan et al., 2014). Figure 4 shows the testing model of the vehicle, along with the transfection results. Both vehicles used existing biological structures and proven molecules that can cross the BBB to complex with their drugs through modifications and deliver to the affected area successfully.

In a study by Janowicz et al., PEGylated hyperbranched polymers to deliver doxorubicin targeted to an ephrin receptor (EphA2) to reduce tumor growth were used to test at what stage of brain cancer nanomedicine was most effective as a treatment (Janowicz et al., 2022). This study was conducted using mouse models exploring how changes in brain anatomy and the BBB that occur during the progression of brain cancer affect delivery of nanomedicines to affected areas. As cancer progresses, the BBB becomes leaky, which can also cause medication delivery to become more complicated (Arvanitis et al., 2020). The study found that delivering nanomedicines at the early stages of leakiness lead to the most effective release of the drug and treatment of the disease.

Other avenues for glioblastoma therapeutics investigate the exploitation of native processes in the body, including specific materials and receptors to treat glioblastoma. Lu et al. studied the use of a self-assembled complex of hemoglobin, lactate oxidase, and bis[2,4,5-trichloro-6-(pentyloxycarbonyl)phenyl] oxalate – photosensitizer chlorin e6 (CPPO-Ce6) encapsulated with U251 glioma cell membranes to increase the metabolism of lactate in cancer cells through the delivery of synergistic lactic acid metabolic therapy and chemiexcited photodynamic therapy (PDT) (Lu et al., 2022). This biomimetic complex, called M@HPLC, was studied in both Transwell and mouse models which both showed uptake specifically in U251 Glioma cells, which are rat-derived glioma cells. The NP system had a diameter of around 118.7 nm and had a zeta potential of −29.1 mV. In the Transwell model, there was successful delivery to both endothelial cells and lower glioma cells using the M@HPLC complex. In mice, the drug was found to cross the BBB and deliver the synergistic system successfully. In Ramalho et al., Asiatic acid, a natural compound with anti-tumor properties and low toxicity but poor bioavailability, was delivered using a Transferrin coupled poly(lactic-co-glycolic acid) (PLGA) NP (Ramalho et al., 2022; Nagoor Meeran et al., 2018). The NP showed a mean size less than 200 nm and a negative zeta potential, which indicated that it could interact with the BBB. The transferrin allowed for interaction with specifically BBB cells, which carry the transferrin receptor to allow passage of the compound to the glioblastoma. This use of transferrin to allow a small drug complex to cross the BBB effectively is a technique that can be extended to many CNS targeting drugs (Wiley et al., 2013). Lu et al. describes the use of a graphene oxide based vehicle conjugated with irinotecan (CPT-11) and cetuximab (CET) that allows for pH sensitive and body temperature dependent release of shRNA and CPT-11 into a tumor to induce apoptosis (Lu et al., 2020). The NP exhibited a 279 nm size with a −11.7 mV zeta potential. The vehicle was studied found to show a controlled release in the formed hydrogel and was able to be targeted to specific areas of the cell using antibody conjugation to the CET, as shown in mouse models. These techniques use the natural anatomy of the BBB to deliver drugs to their targeted location in the treatment of glioblastoma.

#### 2.2.2 Cancer therapeutics in clinical trials

There are currently three active clinical trials in progress testing different nanomedicine techniques in the treatment of glioblastomas. One trial is currently in Phase I with the FDA and being run by the University of California, San Francisco. The drug uses a nanoliposome to encase CPT-11 for individuals genetically predisposed to develop one of the various types of glioblastoma multiforme and have previously been diagnosed with it (ClinicalTrials.gov, 2022a). This drug is administered intravenously every 3 weeks until improvement is seen or until there are signs of toxicity. This study is measuring the safety of the drug as well as monitoring for any signs of efficacy in human subjects. The second study, currently in Phase II with the FDA and sponsored by the University of Regensburg, is a PEGylated liposome containing Doxorubicin that is used in conjunction with radiation therapy and TMZ to treat glioblastoma. In vivo models, the liposome was shown to improve the drug’s penetration through the BBB, which makes it beneficial in the delivery of the effective drug doxorubicin across the BBB to help treat the condition. This drug is administered twice a month on the 1st and 15th days and is being monitored longitudinally based on data from completed toxicity studies (ClinicalTrials.gov, 2009). The third study is currently in Phase II as well and uses a PEGylated topoisomerase I inhibitor polymer etirinotecan pegol (NKTR-102) conjugated with CPT-11 to improve the BBB penetration of CPT-11 and increase the inhibition caused by CPT-11. Previous *in vivo* models have found that this polymer ensures that the cancer is exposed to higher doses of the drug and has fewer systemic side effects due to the localization of treatment (ClinicalTrials.gov, 1663).

### 2.3 Parkinson’s disease (PD)

**FIGURE 5:**
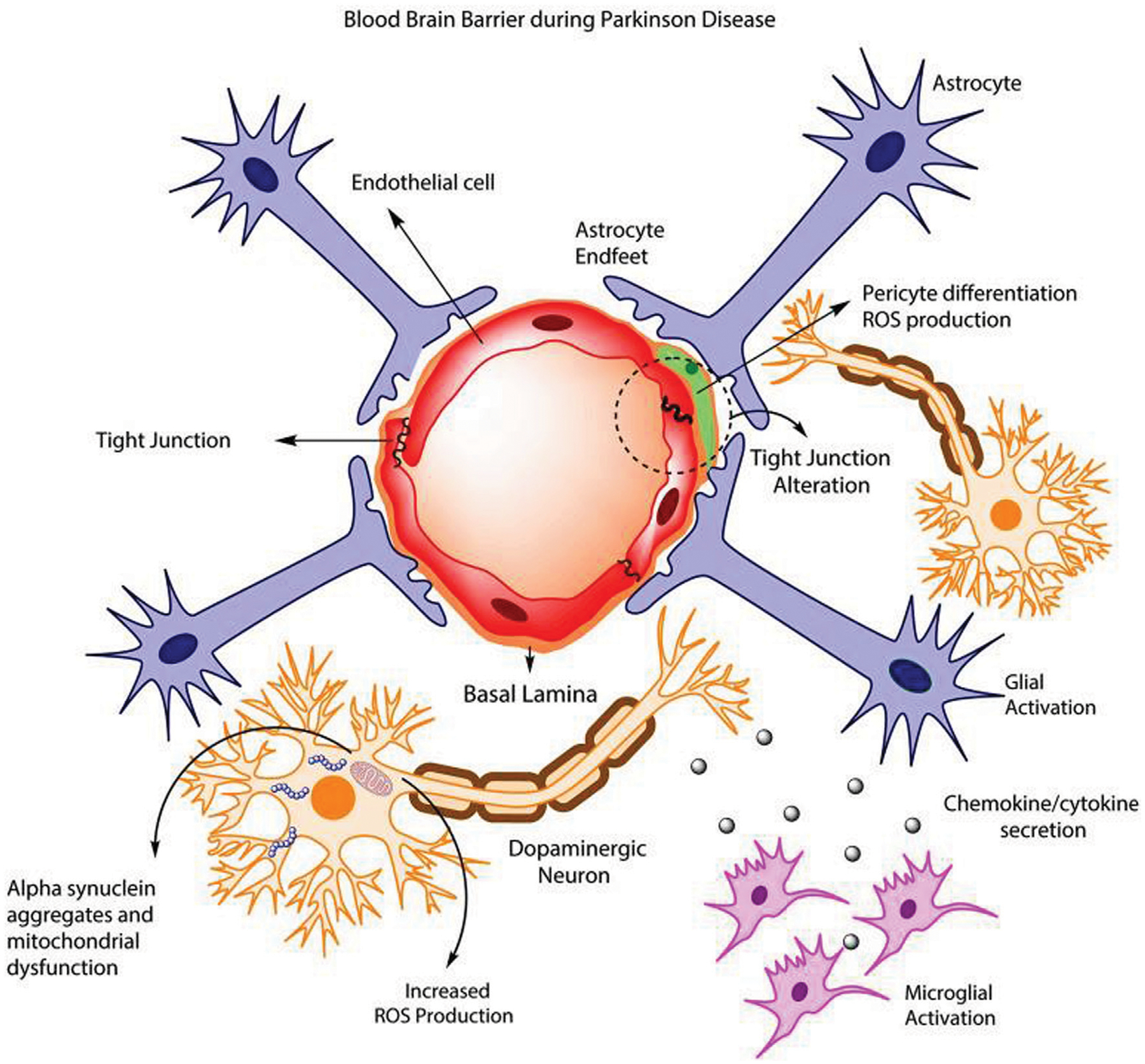
BBB dysfunction during PD schematic. Reproduced from (Cabezas et al., 2014).

Parkinson’s disease is a brain disorder affecting the basal ganglia of the brain that causes uncontrollable shaking, stiffness, and difficulty balancing and coordinating movements. This disease is degenerative and can even progress to difficulty with walking and talking (National Institute on Aging, 2022b). The basal ganglia is a portion of the brain located near the center of the brain and is responsible for managing signals sent by various areas of the brain that affect muscle movement (Cleveland Clinic, 2022). It is also responsible for producing dopamine and many of the nerve endings associated with norepinephrine production, which causes the movement issues seen (José et al., 2023). The cause of the death of these neurons is still unknown, but age, environment, and genetic factors are thought to play a role in the risk of developing this disease (Gorell et al., 2004). PD is also marked by an increase in the amount of alpha-synuclein found in the brain, which is currently being researched to determine its role in disease progression, and which affects BBB physiology as shown in Figure 5 (Rodriguez-Leyva et al., 2017). PD affects more than one million people in the United States (parkinson, 2022). Available treatments for this disease include therapeutic surgery, but mainly include the use of levodopa which is a form of synthetic dopamine (mayoclinic, 2022b). There are also other current research targets, however, based on changes in anatomy in the brain seen in PD patients. NPs are currently being used in this research as a vehicle for these therapeutics to cross the BBB and increase bioavailability.

#### 2.3.1 Current NP-based PD research

**FIGURE 6:**
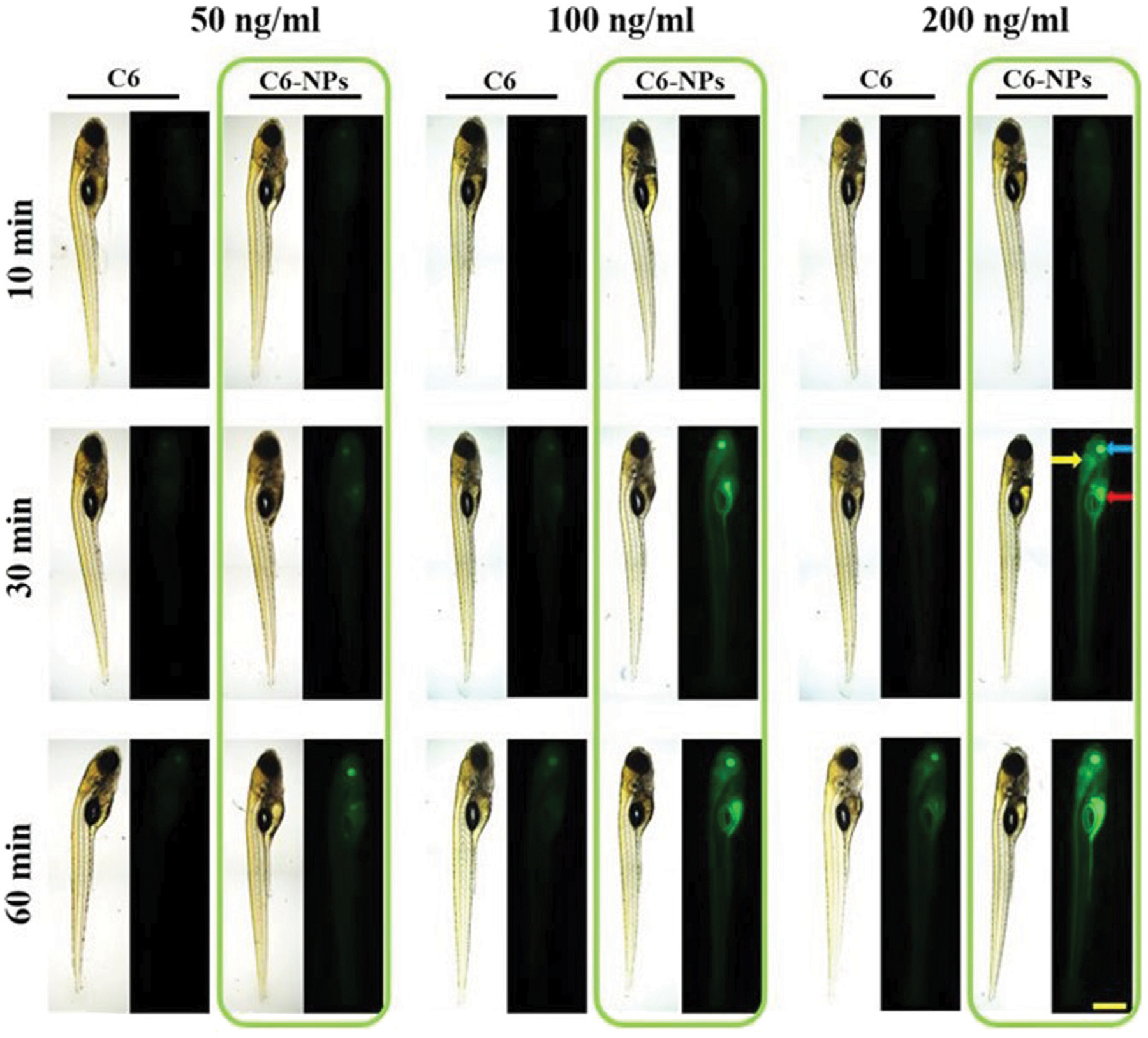
*In vivo* Parkinson’s disease modeling and NP delivery. Observation of BBB penetration using dpf Zebrafish model. Green fluorescence indicates effective cellular uptake of NPs. Reproduced from (Zhao et al., 2020).

Current research investigating nanotherapies for PD centers around the delivery of controlling enzymes and activities known to be associated with PD, as current treatments revolve around dopamine replacement or brain stimulation to compensate for neuron death. One study by Zhao et al. investigates the use of Ginkgolide B, a plant drug thought to be effective against PD, which has poor bioavailability, paired with a poly(ethylene glycol)-copoly(ε-caprolactone) (PEG-PCL) vehicle to help it cross the BBB. Ginkgolide B is a drug that has shown neuroprotective effects in stroke patients, and is a research target for treatment of both PD and AD patients (Mohammad Nabavi et al., 2015). The NP complex had a size of 91.26 nm with a zeta potential of −12.09 mV. In zebrafish models, it was shown that the vehicle helped the drug to have an extended release, which would allow more periodic injections of the drug into the brain during treatment. In mouse models, the drug was successful in crossing the BBB, which was discovered through tissue studies (Zhao et al., 2020). Figure 6 shows the cellular uptake of the vehicle into zebrafish cells which shows the model’s potential use. Another study by Jennings et al. investigated the use of a nanoparticle-based system to inhibit leucine-rich repeat kinase 2 (LRRK2) activity, which would increase lysosome activity that is thought to alleviate symptoms of PD (Jeong and Lee, 2020). The NP used is DNL2101, a selective, ATP-competitive, CNS penetrable LRRK2 inhibitor with formula 2-methyl-2-(3-methyl-4-((4-(methylamino)-5-(trifluoromethyl)pyrimidin-2-yl)amino)-1H-pyrazol-1-yl)propanenitrile. In this case, however, the NP itself is the drug that works to do this and can pass through the BBB, but also targets other tissues such as the kidneys and liver. This drug was found to be somewhat harsh, with some mouse models experiencing kidney and lung issues that were reversible and some toxicity after a prolonged period. However, when tested in humans, it was found that the drug was not toxic in lower dosages and was effective in inhibiting the gene LRRK2 (Jennings et al., 2022).

Joy et al. describe the use of a transdermal delivery system to deliver a complex of Brahmi, a herbal plant thought to have significant nervous system therapeutic effects, and a lipid nanoparticle (Joy et al., 2022). This patch system, made from a combination of polyvinyl alcohol (PVA) and the Brahmi-solid lipid nanoparticle complex, is a dissolvable and painless method of distribution of the NP, which makes patient compliance and treatment more successful, and are able to be manufactured without a cleanroom leading to greater ability to create them at a greater scale to treat more people (Ghanbariamin et al., 2023). The formulation had a zeta potential of −19.21 mV and the NP was in a size range between 120 and 200 nm. Patches in general are very useful for situations that call for targeted drug delivery, such as treatment of osteoarthritis, and therefore may be applied to targeted delivery to the brain (Cao et al., 2021). The lipid NP made the structure of the drug itself more optimal for delivery, which allowed for uptake to the brain as seen in mice. The patch itself was also tested on rat skin and found to have no irritating effect, which means that the patch system would be effective in delivering the medication. This method of delivering an effective lipid NP treatment is important because it indicates a new method of drug delivery to offer to patients.

Wang et al. studied the use of poly(ethylene glycol)-b-poly(trimethylene carbonate) (PEG-PTMC) NPs to deliver Ginkgolide B (Wang Q. et al., 2022). PEG-PTMC is an FDA-approved material that can be used for drug delivery, so using this complex is a method to ensure adequate complexing of the drug to the vehicle. In zebrafish, the NP was found to be highly stable and noncytotoxic. In a mouse model, the drug was successful in delivering across the BBB, which offers another avenue for investigating the use of FDA approved NPs with PD treating medications. In Gao et al., the use of MgOp@PPLP, which is a complex of MgO nanoparticles and a polydopamine shell with polyethylene glycol (PEG), lactoferrin, and puerarin on the surface to improve hydrophilicity and tissue targeting of the drug, with an anti-SNCA plasmid inside, which combats alpha synuclein creation (Gao et al., 2022). These NPs were guided to their target tissue using near-infrared radiation and crossed the BBB, allowing the delivery of gene therapy and antioxidant therapy to the disease source. These treatments offer potential avenues of investigation for various complexes that drugs can be paired with to effectively deliver across the BBB.

#### 2.3.2 PD therapeutics in clinical trails

There are two nanoparticle-based treatments for Parkinson’s disease that are currently in the clinical trial phase of testing. One drug is developed by InnoMedica Schweiz AG and tests the replacement of ganglioside GM1 in PD patients with a liposome system for successful delivery (Maria et al., 2022). The drug is administered via dose escalation leading up to the maximum acceptable dose. GM1 has previously been found to be a neuroprotective agent that helps fundamental functional processes in neurons, especially in PD patients, so this study aims to explore how this drug can be safely administered, effectively delivered, and how effective it is compared to Levodopa, which is the current treatment (ClinicalTrials.gov, 2022b). Another drug currently in clinical trials for use in treating PD is a LRRK2 inhibitor drug DNL2101 produced by Biogen, Inc. in collaboration with Denali therapeutics. It uses a small molecule inhibitor of LRRK2 to increase lysosome activity in PD patients, relieving symptoms. This drug is administered by mouth four times daily and the study is being conducted longitudinally to explore its long term effects (Jennings et al., 2022).

### 2.4 Multiple sclerosis (MS)

Multiple sclerosis is a chronic autoimmune disease affecting the nerve fibers and myelin sheaths of nerves in the CNS. Immune cells attack these healthy tissues, leading to inflammation that alters the messages sent to the brain (BFGh et al., 2013). Since myelin helps messages travel to the brain faster from the extremities, in MS patients, these messages may be slowed or blocked entirely. Most patients when initially diagnosed have relapsing-remitting MS, or a form of the disease where patients are mostly symptom-free, but have flares of the disease at unpredictable times, which can cause a variety of symptoms (Shepherd Center, 2023). These include fatigue, numbness, tingling, blurred vision, weakness, and unsteady gait. The extent that these symptoms progress depends on the patient and their case. For most patients, symptoms of MS increase over time from flare to flare, displaying the symptoms of progressive multiple sclerosis (McNamara, 2022). The underlying cause of multiple sclerosis is unknown, but a possible trigger could be the Epstein-Barr virus, which is a human virus in the herpes family (Bjornevik et al., 2022). Women are more likely than men to develop MS and onset is usually between the ages of 20 and 40. Risk factors include being of northern European descent, having a family history of MS, living in a temperate climate, having low levels of vitamin D, having other autoimmune diseases, and smoking (Mayo Clinic, 2022). It is estimated that more than 2.8 million people worldwide have MS (singlecare, 2022). The current treatments for multiple sclerosis include treating MS flares with steroids, treating MS symptoms, immunosuppressive drugs, and dimethyl fumarate (which is FDA-approved) (webmd, 2022). Current nanoparticle research focuses on delivering dimethyl fumarate across the blood-brain barrier to increase the efficacy of the drug in the target tissue and increase the bioavailability of the drug as well.

#### 2.4.1 Current NP-based MS research

Current research in the use of nanotherapeutics for the treatment of MS focuses on methods to effectively deliver already FDA approved treatments, such as dimethyl fumarate (DMF) (Bomprezzi, 2015). A study by Mehdi et al. looked at the use of modified platelets to deliver DMF through the BBB, as DMF currently has relatively low bioavailability. The zeta potential of this complex was +7 mV, and the size of 118.33 nm was ideal for successful transfection. This study found that the positive charge of the complex was key to successful interaction with endothelial cells which led to successful transcytosis (Mehdi-almadarlou et al., 2022). Another study by Sinha et al. used chitosan-alginate core-shell-corona shaped NPs to deliver DMF for long-term release, which had negative zeta potential of −27.2 mV and a bigger size of 561 nm, which lended it a larger surface area that was ideal for the matrix delivery system being investigated. This study, however, did not focus on the BBB and instead conducted various dissolution tests of the NP system to determine the best way to create an effective slow-release drug (Sinha et al., 2021). These methods both show that existing therapies can also be improved using NP systems to deliver across the BBB more effectively.

Other therapies for MS focus on blocking the body’s response to the disease, which includes the activation of the immune system against native cells, causing the breakdown of myelin sheaths (Fabrizio and Mario, 2005). In Tredicine et al., the use of liposomes was explored to block the function of T cells that attack myelin in the nervous system by downregulating Th17 activation using a peptide from myelin basic protein to reduce inflammation, promoting T regulatory lymphocytes, downregulating Th1 and Th17, and interfering with the pro/anti inflammatory macrophage balance (Tredicine et al., 2023). This was done using a liposome system, which was made from a double layer of phosphatidylserine, which was able to interact with the markers to achieve downregulation as tested in mice. The NP was a spherical bilayer with a size of 298.51 nm and a zeta potential of −38.87 mV, which showed transfection in monocytes and T cells from MS patients and in mouse models. Another study by Triantafyllakou et al., studied using a PLGA NP encapsulating a myelin peptide from the myelin oligodendrocyte glycoprotein epitope (rMOG) complexed with glucosamine to target antigen presenting immune cells and prevent self-attack (Triantafyllakou et al., 2022). Specifically, this NP is able to bind to dendritic cells and prevent them from inducing additional autoimmune activity. The NP had a spherical morphology with a size of 378.4 nm and a zeta potential of −34.8 mV. This system was tested in both *in vitro* and *in vivo* models on mice with experimental autoimmune encephalitis (EAE) which is thought to bear many similarities to MS in the autoimmune triggers. Results showed that there was significant suppression of disease symptoms in treated mice and that the glycosylation played a role in the efficacy of the complex. A study by Titus et al. studied the use of digoxin, which is a common medication used to treat heart issues by inhibiting the sodium-potassium pump and thought to have pro-myelinating properties, in combination with a poly(lactide-co-glycolide) (PLG) NP complexed with autoantigens to both prevent autoimmune attack and stimulate the regeneration of myelin-producing cells, oligodendrocytes, in the CNS (Titus et al., 2022). The NP was of spherical morphology and had a mean size of 483.7 nm with a charge of −84.7 mV. This regimen was tested *in vivo* in mouse models and was studied both for efficacy in delivery, measured with flow cytometry, and for any alterations made to the makeup of the BBB due to its targeting. The targeting was found to be sufficient to the brain with no specific impact of the makeup of the BBB, which makes this a viable treatment. The NPs investigated in these studies provide viable treatment plans for MS and are potential avenues for future drugs that target the pathophysiology of the disease.

## 3 Challenges and future directions

BBB transport systems require the ability to both be small and carry a certain charge while complexing with a drug, which can be difficult to overcome depending on the intended treatment purpose and the drug itself. These challenges can be overcome through creative solutions, including using new technology that allows for different conformations in different conditions, which can be used to improve selectivity and masking of agents to be able to cross the BBB. As these treatments are developed, they can be applied to more and more diseases through the surface functionalization of nanoparticles and complexing with existing drugs, which will improve many lives. Rare diseases have very limited options currently, but nanomedicine opens a new door for these treatments. Nanomedicines may offer an option that requires fewer research dollars to be able to combat these devastating diseases effectively despite the smaller population affected.

A new treatment method to deliver particles across the BBB is Janus-based nanotubes (JBNTs), which mimic the structure of DNA but are able to carry a charge and vary their size depending on side chains of the structure (Landolina et al., 2022). Each side chain or modification added to these nanotubes adds to the function of these structures, both in targeting and in treatment capability of the specific condition application (Yau et al., 2019b). These materials self-assemble using the same principles as DNA to create a nanotube that is flexible enough to complex with RNAs targeted to a variety of different tissues, but mimic natural collagen to avoid potential immune response (Zhang et al., 2008). When compared with the similar carbon nanotube, JBNTs offer more functionality because of their ability to bond together into larger delivery vehicles and create a variety of different geometries depending on the chemistry of the drug (Griger et al., 2022). Because they are DNA-inspired, JBNTs are also ideal because they avoid many toxic side effects that are associated with current vehicles, including polymers, carbon nanotubes, and liposomes, and demonstrate enhanced endosomal escape (Zhang and Chen, 2019; Lee et al., 2021). Previously, JBNTs have shown success in treating conditions of the musculoskeletal system, including arthritis and cartilage regeneration and damage (Zhou et al., 2021a; Yau et al., 2021b). To increase bone growth around orthopedic implants, JBNTs have shown success *in vitro* to deliver peptides that encourage this growth in osteoblasts and for cartilage scaffold delivery regeneration, especially when paired with adequate supplements to make the environment of the damage ideal for repair (Chen and Webster, 2009; Qihai et al., 2018). Their structure also allows for their use as slow drug release vehicles, which has been demonstrated using the anti-inflammatory drug dexamethasone (Chen et al., 2011). They have also been used to enhance the use of hydrogels in cartilage tissue engineering to deliver cells more efficiently and encourage the adoption of an artificial matrix (Chen et al., 2010). Beyond their applications in orthopedics, they also have been shown to be able to form a nano matrix that recruits mesenchymal stem cells effectively because of their ability to mimic collagen fibers (Zhou et al., 2020d). They also are able to capably deliver mRNA to a target tissue by navigating through the extracellular matrix because of their small size and have been used in models of gene therapy and delivery (Sands et al., 2020; Chen et al., 2016; Zhang et al., 2021). Another application of JBNTs is their ability to form a scaffold for a variety of tissue constructs controlled layer by layer for maximum customization (Zhou et al., 2021b). Due to their high customizability and their demonstrated ability to deliver hydrophobic drugs into densely packed areas, JBNTs are an ideal vehicle for the delivery of mRNA and other therapeutics across the BBB.

Nanomedicine is an area of clinical medicine that is rife with directions for drug development. Many of these new systems lie in research ideas that are inspired by the natural machinery of the body, which helps to avoid immune response and system toxicity. For the treatment of neurodegenerative disease, the best option for treatment is nanomedicine because of the small size and adaptability of these vehicles which allow for adaptable crossing of the BBB and subsequent targeting of the drug to diseased tissues. These systems show that there are a variety of approaches available and still more to be discovered in the treatment of these diseases, both based in FDA-approved vehicles and new vehicles that are the product of new research directions.

## 4 Conclusion

From this review, current treatments in development for major diseases of the CNS were explored and highlighted, focusing on the vehicle used to deliver the drug across the BBB and into the area targeted for treatment. The use of polymers and liposomes as the main vehicles were especially prevalent in the field today because these are systems that are known to work to cross the BBB and therefore can be modified effectively to complex with a variety of different drugs and get them across the BBB to treat the disease. Table 2 summarizes the many vehicles that are being tested for delivery to the brain to treat this variety of CNS diseases. It is important to note that many of these treatments are still in preliminary stages of development, such as testing in Transwell or mouse models which have yet to progress to clinical trial phases. However, many of these diseases also have clinical trials in progress exploring these treatments in human subjects, which offers a hope for the future of treating these degenerative diseases.

## Figures and Tables

**TABLE 1:** Summary of common side chains and their purpose in nanoparticle surface functionalization.

Common side chain names	Purpose in nanoparticle
Polyethylene glycol (PEG) coating	Helps NPs avoid adherence to proteins and removal by macrophages within the body
Targeting ligands	Encourages NP interaction with a single type of cell based on ligand interaction
Poly(lactic-co-glycolic acid) (PLGA)	Used in delivery of antigens and sustained release in a matrix
Polyvinyl alcohol (PVA)	Prevents aggregation of NPs and prevents oxidation of NPs
Transferrin (Tf)	Helps NPs cross the BBB due to Tf expression on the surface of endothelial cells

**TABLE 2:** Summary of therapeutics being investigated for use in treatment of neurodegenerative diseases.

Ref number	Nanoparticle type, size, and charge	Disease target	Therapeutic Payload	Pathology target	Model type	Stage of development
Zhou et al. (2020c)	Polymeric: GalPEG-b-P(Gu) 118 nm	AD	siRNA interfering with BACE1	Block BACE1 enzyme which increase Aβ	*In vitro*: Transwell	Pre-clinical research
					*In vivo*: mouse model	
Arora et al. (2021)	Liposome: GLUT1 targeting ligand mannose and cell penetrating peptide with PEG conjugation 172±3.09 nm + 19±0.9 mV	AD	Gene encoding ApoE2	Upregulate ApoE2 production to decrease Aβ	*In vitro*: Transwell monolayer and coculture	Pre-clinical research
Cesur et al. (2022)	Exploded microbubbles with PVA, BiFeO_3_, Donepezil 164 nm	AD	Donepezil	Administer donepezil using a voltage sensitive NP	*In vitro*: Microfluidic model	Pre-clinical research
Ribeiro et al. (2022)	Mesoporous silica nanocontainers storing curcumin 158.10±9.64 nm −16.73±1.91 mV	AD	Curcumin	Administer curcumin for anti-inflammatory and anti-amyloid properties	*In vitro*: microfluidic model	Pre-clinical research
					*In vivo*: mouse model	
(Andrew et al., 2020)	Liposome: DSPE-DOTA-Gd	AD	Contrast agent	Target anatomical differences in AD patient’s brains	Clinical Trial: Patients with AD	Clinical Trials with Alzeca Biosciences, Inc
Guo et al. (2020)	Protein NP: thiolated paclitaxel-oligo with angiopep-2 units	Glioblastoma	Chemotherapies	Only assembles in altered pH of tumor microenvironment	*In vitro*: Fluorescence cell models	Pre-clinical research
					*In vivo*: mouse models	
Wang et al. (2022a)	Polymeric: PEGylated pH sensitive pMPC-co-(Mmfu-pPEG-MA) 45 nm	Glioblastoma	Antiprogrammed death ligand 1 (anti-PD-L1)	Targets altered pH of tumor microenvironment	*In vitro*: Transwell model	Pre-clinical research
					*In vivo*: mouse model	
Tan et al. (2020)	Polymeric: iRGD guiding peptide with TMZ in PEG-PDPA micelle 71±15 nm 4.3±2.8 mV	Glioblastoma	TMZ, Mn, O_2_	Releases chemotherapy directly to tumor microenvironment to induce apoptosis and oxidative stress	*In vitro*: Transwell model	Pre-clinical research
					*In vivo*: mouse model	
Janowicz et al. (2022)	Polymeric: PEGylated hyperbranched polymers	Glioblastoma	Doxorubicin	Targets leaky BBB to cross through and target tumor	*In vivo*: mouse model	Pre-clinical research
Lu et al. (2022)	U251 Glioma cell membranes with Hb, LOX, CPPO-Ce6 complex 118.7 nm −29.1 mV	Glioblastoma	Lactic acid metabolic therapy and Ce6	Use native cells to increase BBB uptake and transcytosis	*In vitro*: Transwell	Pre-clinical research
					*In vivo*: mouse model	
Ramalho et al. (2022)	Polymeric: Transferrin coupled PLGA <200 nm Negative charge	Glioblastoma	Asiatic acid	Deliver antitumor compound directly to tumor using transferring receptor	*In vitro:* dye release assay	Pre-clinical research
ClinicalTrials.gov (2022a)	Liposome: encases CPT-11 to induce tumor apoptosis	Glioblastoma	Irinotecan (CPT-11)	Delivers gene to individuals at risk for cancer to alter expression	Clinical trial: on selected patients	Clinical Trials with University of California, San Francisco (Phase I)
(ClinicalTrials.gov, 2009)	Liposome: PEGylated vehicle	Glioblastoma	Doxorubicin	Used with radiation to treat cancer	Clincal trial: on selected glioblastoma patients	Clinical Trials with University of Regensburg (Phase II)
ClinicalTrials.gov (1663)	Polymeric: PEGylated vehicle	Glioblastoma	NKTR-102 and CPT-11	Affects topoisomerase to slow down DNA replication in cancer cells	Clinical trial: on selected glioblastoma patients	Clinical Trials with Stanford University (Phase II)
Zhao et al. (2020)	Polymeric: PEG-PCL 91.26±1.34 nm −12.09±0.97 mV	PD	Ginkgolide B	Administer Ginkgolide B which is thought to be effective against PD	*In vivo*: zebrafish models	Pre-clinical research
					*In vivo*: mouse models	
Jennings et al. (2022)	Therapeutic NP (no drug complexed)	PD	DNL2101	Inhibits LRRK2 activity to increase lysosome activity	*In vivo*: mouse models	Clinical Trials with Biogen Inc. and Denali Therapeutics
					*In vivo*: human subjects (Clinical Trial)	
Joy et al. (2022)	Liposome complexed with *B. monnieri* and polyvinyl alcohol −19.21 mV	PD	*B. monnieri* (Brahmi)	Administers Brahmi transdermally for nervous system therapy	*In vivo*: mouse model	Pre-clinical research
					*In vivo*: rat model	
Wang et al. (2022b)	Polymeric: PEG-PTMC	PD	Ginkgolide B	Administers Ginkogolide B for therapeutic effects against PD	*In vivo*: zebrafish models	Pre-clinical research
	Around 90 nm				*In vivo*: mouse models	
	Around 15 mV					
Gao et al. (2022)	Polymeric: MgO and polydopamine encapsulating drug MgOp@PPLP	PD	Anti-SNCA plasmid to fight alpha synuclein formation	NP targets tissue and prevents hydrophobicity from interfering with delivery	*In vitro*: Transwell model	Pre-clinical research
					*In vivo*: mouse models	
ClinicalTrials.gov (2022b)	Liposome: GM1 complexed with liposome	PD	Ganglioside GM1	GM1 has neuroprotective properties in PD so administration is an obstacle	Clinical trial: selected PD patients	Clinical Trials with InnoMedica Schweiz AG
Mehdi-almadarlou et al. (2022)	Modified platelets complexed with DMF 118.33 nm + 7mV	MS	Dimethyl fumarate	Lower lymphocyte profile in MS patients to reduce inflammation	*In vitro* and *in vivo*	Pre-clinical research
Sinha et al. (2021)	Chitosan-alginate core-shell-corona shaped NPs 561±53.05 nm −27.2±6.33 mV	MS	Dimethyl fumarate	Release DMF over a long period of time	Dissolution tests *in vitro* for slow release	Pre-clinical research
Tredicine et al. (2023)	Liposomes from double layer of phosphatidylserine	MS	Peptide from myelin basic protein	Interact with T cell markers to downregulate Th17 inflammatory response	*In vitro*: Transwell model	Pre-clinical research
	298.5±57.27 nm				*In vivo:* mouse models (EAE)	
	−38.87±1.72 mV				*In vivo*: human MS patients	
Triantafyllakou et al. (2022)	Polymeric: PLGA NP encapsulating myelin peptide with glucosamine 378.4 nm −34.8 mV	MS	Peptide from myelin oligodendricyte glycoprotein epitope	Prevents dendritic cells from inducing more autoimmune activity and attack antigen presenting cells	*In vitro*	Pre-clinical research
					*In vivo:* EAE induced mouse models	
Titus et al. (2022)	Polymeric: PLG NP complexed with autoantigens and sodium digoxin 483.7 nm −84.7±14.3 mV	MS	Digoxin	Interacts with BBB to allow successful transfection and inhibits sodium-potassium pump	*In vivo*: mouse models	Pre-clinical research
